# Silver-doped phosphate coacervates to inhibit pathogenic bacteria associated with wound infections: an in vitro study

**DOI:** 10.1038/s41598-022-13375-y

**Published:** 2022-06-24

**Authors:** Athanasios Nikolaou, Monica Felipe-Sotelo, Robert Dorey, Jorge Gutierrez-Merino, Daniela Carta

**Affiliations:** 1 Department of Chemistry, University of Surrey, Guildford, GU27XH UK; 2 School of Biosciences and Medicine, University of Surrey, Guildford, GU27XH UK; 3 School of Mechanical Engineering Sciences, University of Surrey, Guildford, GU27XH UK

**Keywords:** Antimicrobial resistance, Biomaterials

## Abstract

There is a great demand from patients requiring skin repair, as a result of poorly healed acute wounds or chronic wounds. These patients are at high risk of constant inflammation that often leads to life-threatening infections. Therefore, there is an urgent need for new materials that could rapidly stimulate the healing process and simultaneously prevent infections. Phosphate-based coacervates (PC) have been the subject of increased interest due to their great potential in tissue regeneration and as controlled delivery systems. Being bioresorbable, they dissolve over time and simultaneously release therapeutic species in a continuous manner. Of particular interest is the controlled release of metallic antibacterial ions (e.g. Ag^+^), a promising alternative to conventional treatments based on antibiotics, often associated with antibacterial resistance (AMR). This study investigates a series of PC gels containing a range of concentrations of the antibacterial ion Ag^+^ (0.1, 0.3 and 0.75 mol%). Dissolution tests have demonstrated controlled release of Ag^+^ over time, resulting in a significant bacterial reduction (up to 7 log), against both non-AMR and AMR strains of both Gram-positive and Gram-negative bacteria (*Staphylococcus aureus*, *Enterococcus faecalis*, *Escherichia coli* and *Pseudomonas aeruginosa*). Dissolution tests have also shown controlled release of phosphates, Ca^2+^, Na^+^ and Ag^+^ with most of the release occurring in the first 24 h. Biocompatibility studies, assessed using dissolution products in contact with human keratinocyte cells (HaCaT) and bacterial strains, have shown a significant increase in cell viability (*p* ≤ 0.001) when gels are dissolved in cell medium compared to the control*.* These results suggest that gel-like silver doped PCs are promising multifunctional materials for smart wound dressings, being capable of simultaneously inhibit pathogenic bacteria and maintain good cell viability.

## Introduction

Wound management is a major area of global unmet need. Growing elderly and diabetic populations are leading to a sharp increase in the cases of chronic wounds (diabetic foot, venous, and pressure ulcers). A large proportion of these wounds are resistant to current treatments, remaining unhealed for months or years, making chronic wounds a substantial economic, social and clinical challenge^1^. Chronic wounds are normally associated with the proliferation of antibacterial resistance (AMR) bacteria^2,3^, resulting in prolonged antibiotic treatments and the subsequent increase in healthcare costs^4^. AMR is an additional growing global issue that poses a serious threat to public health^5^. In addition, increasing numbers of acute wounds result in surgical site infections, which significantly delay healing. Therefore, there is an urgent need for novel materials that are able, not only to assist with skin healing and regeneration but also to display efficient antibacterial activity.

The demand for wound treatment is extremely high, with the wound care market expected to reach $27.8bn by 2026 worldwide (compound annual growth rate 7.6%)^6^. Therefore, a material capable of promoting tissue regeneration and managing wound infection represent a transformative alternative to conventional dressing-based wound management (physical protection/antibacterial activity). Polyphosphate-based coacervate (PC) gels have been recently investigated as materials with a great potential in hemostasis applications, playing an important role in the coagulation cascade and decreasing blood coagulation^7^. Amorphous polyphosphates have also been shown to enhance wound healing when used in combination with collagen^8^. Moreover, in vivo studies have shown acceleration of wound closure in the presence of polyphosphate nanoparticles^9,10^. Being resorbable, PC are ideal controlled delivery systems, being capable of releasing therapeutic species in the physiological environment in a controlled, continuous, and timely manner. PC therefore have the potential to be multifunctional smart biomaterials, being able to induce simultaneously tissue healing/regeneration and release of therapeutic species to reduce bacterial infection^7^.

PC are prepared via the simple, sustainable route of coacervation^11^. This method consists of the slow addition of an aqueous solution of divalent ions (e.g. Ca^2+^, Mg^2+^, Mn^2+^) to an aqueous solution of sodium polyphosphate at room temperature. Upon addition, a phase separation occurs with the formation of a viscous, opaque lower layer (PC) and an upper aqueous layer. Following the removal of the upper layer, the as-made, gel-like PC is isolated. The synthesis of PC is very versatile, and a variety of therapeutic species, including thermal sensitive, can be added to the material. Although the polyphosphate coacervation process is well known, only recently has gained renewed attention due to its potential applications in biomedicine^12^.

Most of the PC studies to date focus on their synthesis^12^, thermal properties^7^, rheology^13^, and change of properties over time^7^. Some studies have investigated dried PC as bulk glass powders^7^ and fibres^11^ in terms of structure and antibacterial activity when doped^7,14,15^ with Ag^+^ or Cu^2+^. However, to the knowledge of the authors, the antibacterial capabilities of PC in gel form have not been explored to date. This work investigates for the first time the antibacterial effects of PC in gel form when doped with various amounts of the antibacterial metallic ion Ag^+^.

This work is in line with the current trend in antibacterial materials research that leads towards finding alternative non-antibiotic therapies such as treatments based on antibacterial metallic ions^16^ and metallic nanoparticles^17^, less prone to develop AMR. The versatility of the in-solution coacervation method allows the inclusion in the coacervate gel of a variety of antibacterial metallic ions such as Ag^+^, Cu^2+^, Zn^2+^, Ga^3+^ and Ce^4+^, which can then be released in a controlled manner in the wound site^18,19^. Previous studies have reported that Ag^+^ embedded in phosphate-based bulk glasses prepared using the traditional melt-quenched route, successfully reduced bacterial viability of *S. aureus* and *P. aeruginosa*^14,20^. Moreover, silver-doped coacervates dry glasses have also shown antibacterial activity against *S. aureus*^14^. The antibacterial activity was mainly ascribed to the dissolution properties of phosphate-based glasses, which can release Ag^+^ ions in a sustainable way. It has to be noted that in this study, we consider the PC in gel form before calcination, and not in its glass state obtained after drying the coacervate gel.

Here we present, a series of silver-doped PC gels doped with various amounts of Ag^+^ (x = 0.1, 0.3 and 0.75 mol%). The antibacterial efficacy was tested by exposing bacteria commonly associated with wound infections, such as *Staphylococcus aureus* (*S. aureus*), *Enterococcus faecalis* (*E. faecalis*),* Escherichia coli* (*E. coli*) and *Pseudomonas aeruginosa* (*P. aeruginosa*) to the dissolution products of the PC in water and cell medium. For every microorganism, both non-AMR and AMR strains were used. The biocompatibility of silver-doped PC gels on human keratinocyte cells (HaCaT) in combination with bacteria was also investigated. This study is of particular interest given that there is still some controversy on the cytotoxicity of Ag^+^, particularly dependent on its concentration^21,22^.

## Materials and methods

### Synthesis

The following chemicals have been used without further purification; sodium polyphosphate (Na(PO_3_)_n_, Merck), calcium nitrate tetrahydrate (Ca(NO_3_)_2_·4H_2_O, Acros, 99.0%), and silver nitrate (AgNO_3_, Alfa Aesar, 99.9%). 20 mL of 2 M calcium nitrate tetrahydrate were slowly added to an equal volume of 4 M sodium polyphosphate using a syringe pump (20 mL h^−1^) under stirring for 1 h. Upon stirring, phase separation occurred in which an upper aqueous layer separated from a lower opaque coacervate layer. To prepare the 0.1, 0.3, 0.75 mol% Ag^+^ doped samples, 0.083,0.25 and 0.58 mL of a 2 M silver nitrate aqueous solution were added dropwise to the phase-separated mixture, respectively. After the addition of all reactants, the mixtures were stirred for a further hour and the samples were covered and allowed to settle overnight at room temperature. The top aqueous layer was then removed, and the bottom coacervate layer was transferred into a glass vial. Undoped coacervate gels will hereafter be named as PC and silver doped coacervate gels will be hereafter named as PC_AgX, where X is the mol% of Ag^+^.

### Dissolution and pH studies

To assess the chemical species released upon dissolution, 10 mg of each coacervate gel were immersed in 10 mL of deionized water and left in the solution for 1, 3, 5, and 7 days. Three replicates for each condition (n = 3) were performed. The resulting suspensions for each time point were centrifuged at 4800 rpm for 10 min to separate the remaining coacervate gel from the solution. The pH upon dissolution of the gel PC was monitored both in water and cell medium at days 1, 3, 5 and 7 (Mettler Toledo SevenCompact™ pH meter).

Samples were filtered with a 0.45 µm unit (Millipore filter unit, Millex™-GP) followed by acidification with HNO_3_ (for Trace Metal Analysis from Fisher Chemical; final concentration 2% v/v HNO_3_) prior analysis by microwave plasma atomic emission spectroscopy (MP-AES, Agilent 4210). Calibration standards for P, Ca, Na and Ag were prepared from commercial solutions (SPEX CertiPrep™) in 2% v/v HNO_3_ and measured daily. The linear dynamic ranges and limits of detection (LOD, based on 3 × SD of the blank) were 0–10 ppm and 0.17 ppm, 0–6 ppm and 0.04 ppm, 0–10 ppm and 0.25 ppm, 0–100 ppm and 0.50 ppm, respectively for Na (at 588.95 nm), Ca (at 422.67 nm), P (at 213.618 nm) and Ag (at 546.549 nm). For Ca, Na and P measurements, samples required further 1:100 dilution in 2% HNO_3_, whereas a 1:1 dilution was sufficient for the measurement of Ag in the solubility samples.

### Fourier transform infrared (FT-IR) spectroscopy

FTIR spectra were collected using a Perkin Elmer spectrometer 2000-FTIR in the range of 4000–500 cm^−1^ with a resolution of 8 cm^−1^ (32 scans per sample).

### Particle size and zeta potential measurement

A small quantity of each PC gel was dispersed in ultrapure water using ultrasonic agitation for 10 min to produce samples suitable for determining mean particle size and zeta potential of the polyphosphate clusters using dynamic light scattering (Malvern Zetasizer).

### Antibacterial activity

The antibacterial activity of all coacervates was tested against a series of bacterial strains, both non-AMR and AMR, as listed in Table 1. All the strains were cultured in tryptic soy broth (TSB, Oxoid) at 37 °C with shaking at 250 rpm for 16–24 h. For bacterial strains carrying antibiotic-resistant plasmids, the necessary antibiotic concentration was added into TSB. 50 mg of each PC gel were added into 5 mL of the resulting bacterial TSB cultures and incubated at 37 °C under stirring at 250 rpm for 24 h. The undoped (silver-free) coacervate gel was used as a control. After the 24 h-incubation, a drop plate method was used for bacterial colony counting as previously described^23^. Briefly, samples were serially diluted (1:10 fold) under aseptic conditions, and 100 μL-aliquots from each dilution were spread on Tryptic Soya Agar (TSA) plates that were incubated at 37 °C for 24 h. The colonies formed on the plates were counted to calculate the Colony Forming Units per millilitre (CFU/mL) of the sample. The bacterial reduction was finally expressed as log10 CFU/mL with their corresponding error bars representing the standard deviation (two-way ANOVA for each time point). Asterisks illustrate the degree of statistical difference of the samples when compared to the control.

**Table 1:** Bacterial strains used in this study.

Bacterial species	Characteristics^1^	Antibiotics^2^	References
**Non-AMR strains**			
*S. aureus* NCTC 8325	PS47 (36) original strain	none	^24^
*E. faecalis* OG1RF	pMV158-GFP-Tet^R^	Tetracycline^$^ (10 μg/mL)	^25^
*E. coli* K12	Seva231-GFP-Gm^R^	Gentamycin^$^ (10 μg/mL)	^26^
*P. aeruginosa* PAO1	Seva231-GFP-Gm^R^	Gentamycin^$^ (10 μg/mL)	^26^
**AMR strains**			
*S. aureus* NCTC 13656	MRSA (MecA and MupA +ve)	Methicillin and mupirocin*	^24^
*E. coli* NCTC 13351	TEM-3 Extended spectrum beta lactamase	Penicillin derivates*	^24^
*P. aeruginosa* NCTC 13437	VIM-10; VEB-1 +ve	Carbapenems*	^24^
*E. faecalis* NCTC 12201	VanA +ve	Vancomycin*	^24^

^1^Genetic feature of the selected strains.

^2^Antibiotics to which AMR strains are resistant to* and antibiotics used in the cultures of the non-AMR strains^$^ to maintain the expression of the GFP plasmids over time (concentrations indicated in brackets).

### Cell viability studies

Cell viability was determined in the presence of the non-AMR bacterial strains indicated in Table 1. To analyse the toxicological effect of the PCs on skin cells, in vitro biocompatibility tests using HaCaT (human keratinocyte) cells from AddexBio were performed, as previously described^15^. The HaCaT cells were cultured in Eagle’s Essential Medium (ATCC) with 10% v/v fetal bovine serum (Gibco, Invitrogen) and 100 μg mL^−1^ of streptomycin (Thermofisher Scientific, UK) in a humidified incubator at 5% CO_2_ and 37 °C. Cells were routinely passaged on reaching approximately 80–90% of confluence. Simultaneously, 10 mg of each PC gel were immersed in 10 mL of deionised water and cell medium (1:1000) and orbitally incubated at 200 rpm for 24 h at 37 °C. Cells were then seeded into 96-well plates at a density of 5000–10,000 cells per well and incubated with medium overnight before adding 60 µL of the PC dissolution products obtained as described in section “Dissolution and pH studies”. HaCaT cells were challenged with the coacervate dissolution samples but in combination with bacterial cultures at a 1:1 ratio. Deionised water and cell medium were used as controls. After an incubation of 2 days, the media containing the dissolution products (or deionised water) were aspirated and 200 µL of fresh medium were added to each well to measure cell viability based on total mitochondrial dehydrogenase activity. This dehydrogenase activity was determined using the 3-(4,5-dimethylthiazol-2-yl)-2,5-diphenyl tetrazolium bromide (MTT) assay (Sigma-Aldrich). Briefly, 20 µL of a 1:1 MTT phosphate-buffered saline solution were added to each well and incubated at 37 °C and 5% CO_2_ for 3 h. The liquid was aspirated from each well and replaced with 200 µL of dimethyl sulfoxide (DMSO, Sigma-Aldrich) to dissolve the insoluble purple formazan crystals formed within the cells due to the action of the mitochondrial dehydrogenase^27^. The absorbance of the DMSO solutions was measured at 570 nm using a microplate reader (Fluostar-Omega, BMG LABTECH, Bucks, UK). Absorbance was then used to calculate the percentage of cell viability (%) as previously described^28^.

### Antibacterial effect on human keratinocyte (HaCaT) cells

The antibacterial effect of PC gels was further determined in the presence of the non-AMR bacterial strains indicated in Table 1. HaCaT cells were challenged with the coacervate dissolution samples as described above, this time in presence of bacterial cultures (1:1 ratio). The 96-well plates were incubated in a BMG CLARIOStar multi-well plate reader at 5% CO_2_ and 37 °C, and following incubation bacterial growth was recorded based on the expression of absorbance at 600 nm and/or fluorescence emission at 515.20 nm. Fluorescence was only applied to samples containing bacteria expressing the Green Fluorescent Protein (GFP). HaCaT cells not exposed to any PC_Ag samples but exposed to bacteria were used as controls.

## Results and discussion

**Figure 1:**
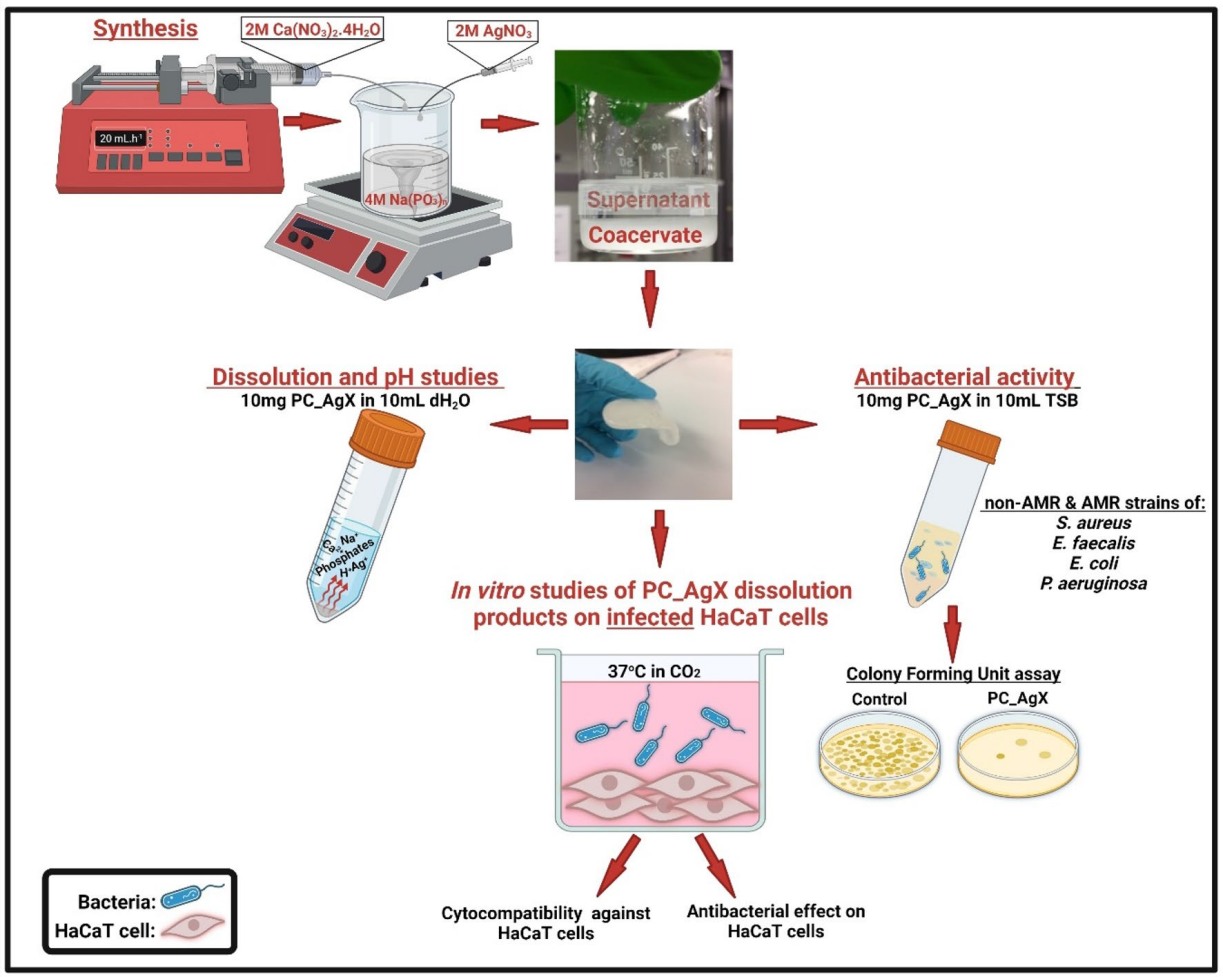
Schematic illustration of the synthesis of the phosphate coacervate gels and main studies performed in the present work.

A schematic illustrating the coacervate process for the synthesis of the PC gels and the studies performed in this work is presented in Fig. 1.

### Synthesis

Sodium polyphosphate (NaPP), an inorganic solid formed by polyphosphate chains, is dissolved in water under stirring. After complete dissolution, a solution of Ca^2+^ is slowly added using a syringe pump (20 mL h^−1^) under constant stirring. Then an appropriate amount of Ag^+^ aqueous solution is added dropwise to the solution above and stirred for an hour to ensure homogenous mixing. Phase separation then occurs with the formation of a viscous liquid immiscible with water (coacervate, bottom layer) and a supernatant aqueous layer. The coacervate in contact with the supernatant layer is left to settle overnight at room temperature. After removal of the supernatant layer, a gel-like coacervate is obtained.

The PC gels have a high water content, and this makes them ideal materials for wound dressings as the moist environment prevents dehydration and facilitates the healing process. This study presents a series of 5 gel samples, containing 0.1, 0.3, 0.75 mol% Ag^+^. All gels were tested for dissolution and pH change in water and cell medium, antibacterial activity against a series of AMR and non-AMR strains and in vitro cytocompatibility studies, as illustrated in Fig. 1.

### Dissolution and pH studies

**Figure 2:**
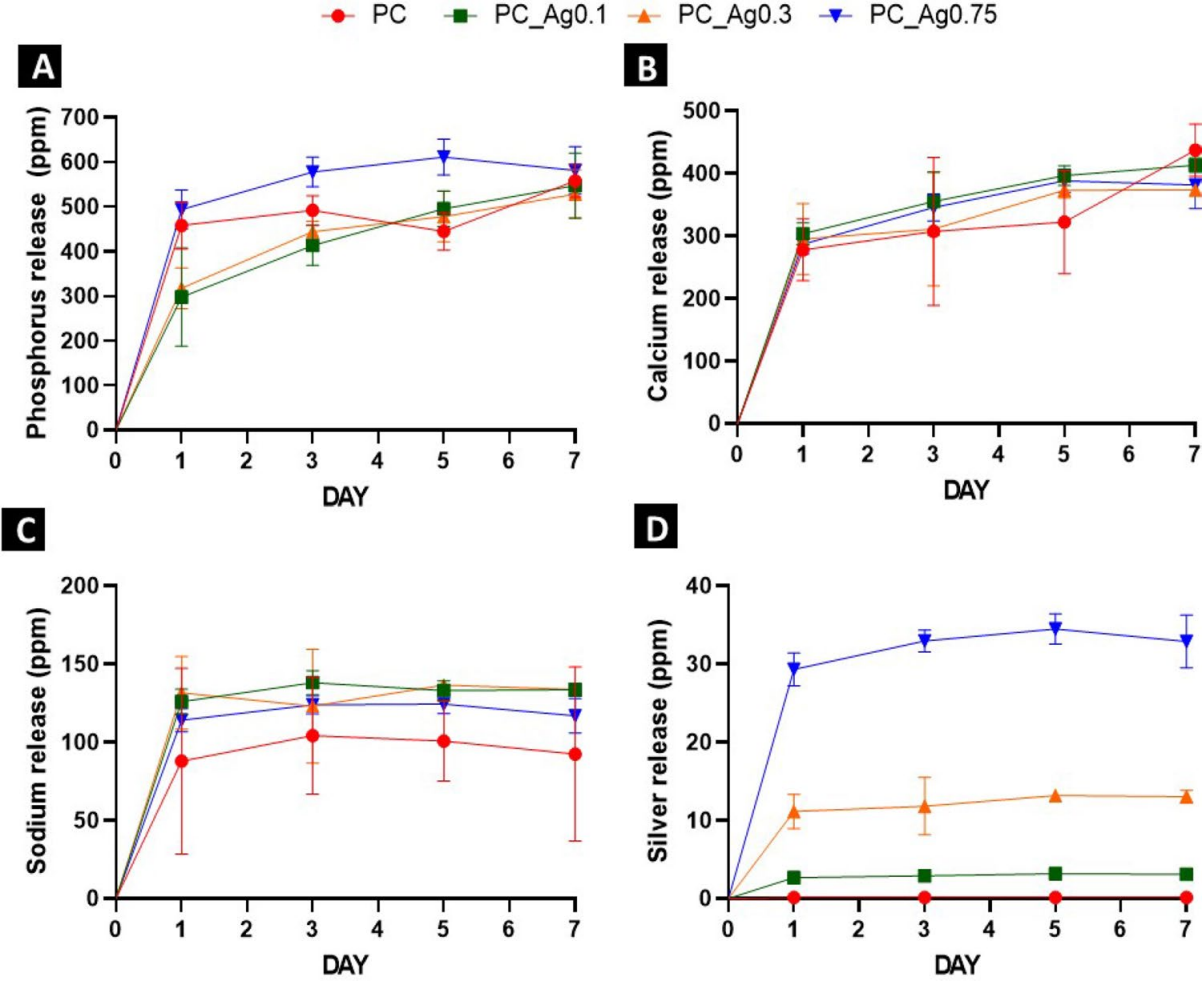
Ion release of (**A**) phosphorus, (**B**) calcium, (**C**) sodium and (**D**) silver in deionized water over 7 days for all PC gels. Error bars indicate the mean ± standard deviation (n = 3).

Dissolution studies were performed by immersing the PC gels in deionized water for up to 7 days. The solutions obtained after 1, 3, 5 and 7 days of immersion were then analysed to quantify the amount of P, Ca, Na and Ag released over time. The resulting dissolution profiles for P, Ca, Na and Ag are indicated in Fig. 2. We observed that, upon degradation, a gradual release of all ions occurs over time, and that most of the P, Ca and Na amounts are released in the first 24 h regardless of the Ag content (300–500 ppm P, 300 ppm Ca, 80–130 ppm Na and 0–30 ppm Ag). As expected, Ag^+^ release increases with the silver content.

**Figure 3:**
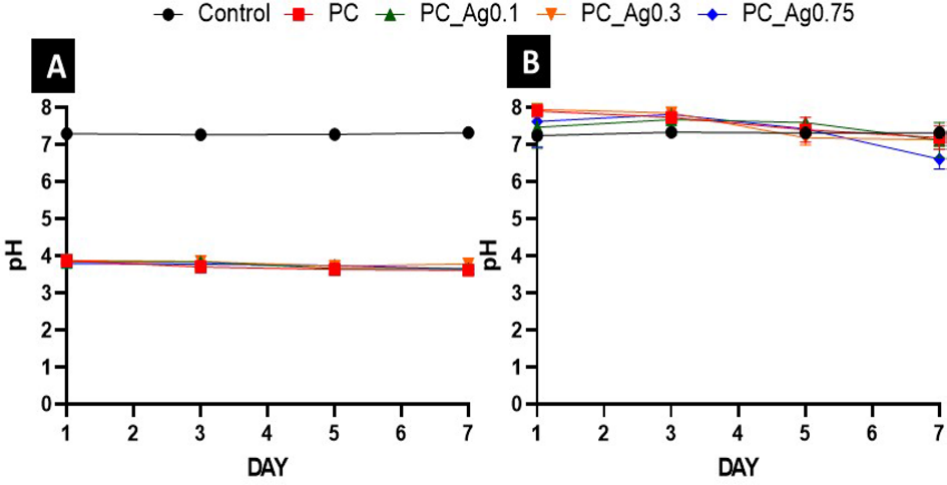
pH analysis of all dissolved in deionised water (**A**) and cell medium (**B**) over 7 days. Error bars represent the SD ± mean of two biological replicates. PBS was used as a control for this experiment.

In addition to the quantification of ions released in deionised water, the pH of the solutions containing the dissolution products was also measured after 1, 3, 5 and 7 days (Fig. 3). Since day 1, the pH of all deionised water solutions was significantly reduced from neutrality (pH ~ 7) to pH ~ 4 (Fig. 3A). After this significant drop, the pH remains relatively stable over the 7 days period, only decreasing slightly below 4. However, when the dissolution is performed in cell medium (Fig. 3B), the initial pH of ~ 8 decreased only slightly over the 7 days, remaining around neutrality (pH ~ 7).

### FT-IR spectroscopy

**Figure 4:**
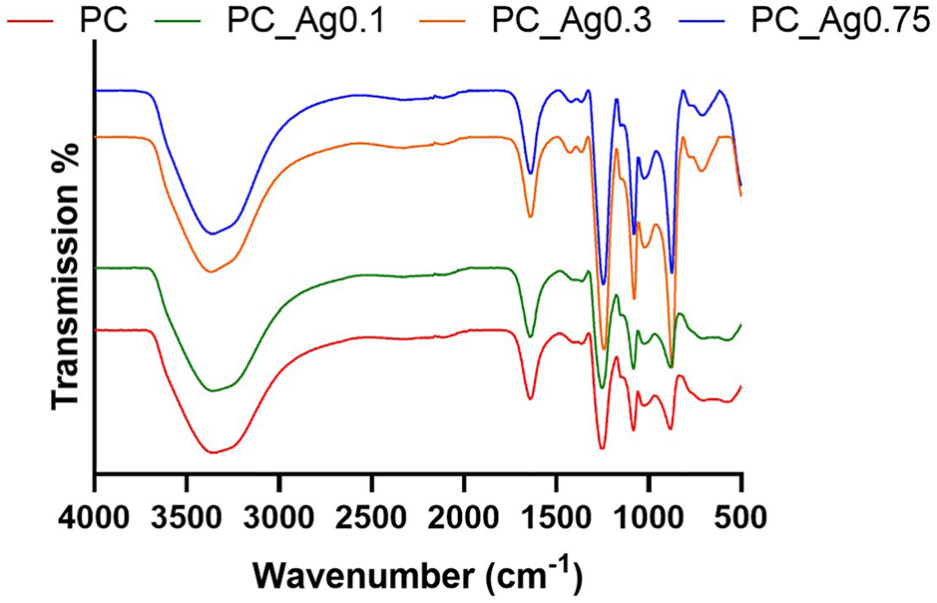
FTIR spectra of the undoped (PC) and silver doped (PC_Ag) gels.

The structure of the phosphate gel network was investigated using FT-IR spectroscopy (Fig. 4). The vibrations observed are similar to those reported for dry glasses^7^. The absorption band near 1250 cm^−1^ is assigned to the asymmetric stretch in the PO_2_ metaphosphate units^29^, *v*_*as*_(PO_2_)^−^. The band near to 1100 cm^−1^ is assigned to the asymmetric stretching of chain-terminating PO_3_ groups^30^, *ν*_as_(PO_3_)^2−^. The absorption band near 900 cm^−1^ is assigned to the asymmetric stretching modes of the P–O–P groups, *ν*_*as*_(P–O–P)^31^. The peak at 540 cm^−1^ is attributed to O–P–O deformation modes^31^. The peaks at ~ 1600 cm^−1^ and 3400 cm^−1^ are assigned to the bending and stretching of O–H bonding in the residual water^15^. No significant differences were detected between the undoped gel and gels doped with different concentrations of silver.

### Dynamic light scattering (DLS) analysis and zeta potential measurement

The average zeta potentials of the different PC gels were found to be between − 4 and − 9 mV with no clear trend exhibited between gels as the difference between samples was comparable to difference between measurements of individual samples. Due to the near-zero zeta potential the particles exhibited a strong tendency to agglomerate. This is as expected given the way in which the gels were manufactured in this study. Due to the tendency of the polyphosphate clusters to agglomerate all dispersions were very unstable and exhibited polydisperse particle size distribution making accurate determination of primary particle size impossible. PC, PC_Ag0.1 and PC_Ag0.3 all exhibited a distribution of particles centred around 130–350 nm. PC_Ag0.75 only exhibited a distribution centred around 1000–2000 nm, but this does not discount the possibility of smaller particles being present as the signal from the large particles is likely to have masked that from the smaller particles. Again, this is a consequence of the very poor stability of the suspension.

### Antibacterial activity

**Figure 5:**
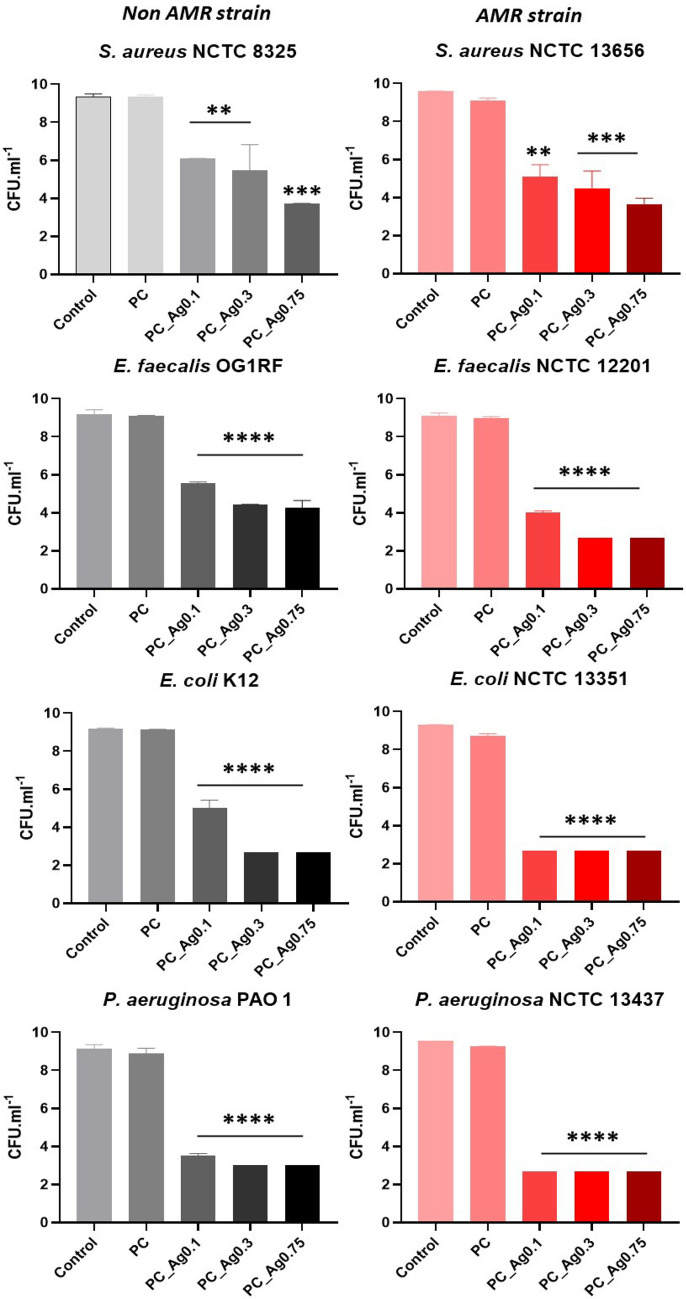
Antibacterial activity of PC gels against non-AMR and AMR bacterial strains of *S. aureus*, *E. faecalis*, *E. coli* and *P. aeruginosa*. Bacterial reduction is expressed as the mean of CFU/mL ± standard deviation (error bars). Statistical analysis was performed using two-way ANOVA (**p ≤ 0.01; ***p ≤ 0.001; ****p ≤ 0.0001). Asterisks illustrate the degree of statistical difference of the samples when compared to the control.

All coacervate samples, undoped and doped with increasing concentrations of silver (0.1, 0.3, 0.75 mol% of Ag), were challenged against bacteria associated with wound infections (*S. aureus*, *E. faecalis*, *E. coli* and *P. aeruginosa*) (Fig. 5). For each sample, antibacterial tests were performed against two different strains, a non-AMR strain (Fig. 5, left) and a strain associated with AMR (Fig. 5, right). In all the experiments, three biological replicates and cultures with no coacervate gel were used as controls. The bacterial reduction was then determined after 24 h of incubation. Similarly to previous studies on phosphate-based glasses prepared by melt quenching and silicate-based glasses^32,33^, the undoped PC samples showed a small, but no significant, antibacterial activity.

The antibacterial activity displayed by the doped gels positively correlates with the Ag^+^ content; the antibacterial activity seems also dependent on the type of bacteria and whether the strains are resistant to antibiotics. In particular, the non-AMR strains are more tolerant than the AMR strains. When bacteria are challenged with PC_Ag0.1, we observed that the log reductions for the AMR strains are significantly higher than that recorded for non-AMR strains. When exposed to PC_Ag0.3 and PC_Ag0.75 the differences between the strains are not that evident and, in some cases, total inhibition is observed for both AMR and non-AMR strains. Another interesting observation is that the Gram-negative bacteria (*E. coli* and *P. aeruginosa*), either non-AMR or AMR strains, are more sensitive to the silver action than the Gram-positive bacteria (*S. aureus* and *E. faecalis*). Compared to the *S. aureus* and *E. faecalis*, the AMR strains of *E. coli* and *P. aeruginosa* were inhibited with all Ag^+^ concentrations, reaching the limit of detection point after 24 h (*p* ≤ 0.0001) with a log reduction of 6.8. Although the non-AMR strains of both *E. coli* and *P. aeruginosa* have shown slightly less sensitivity, by comparison with the AMR strains, significant log reductions (*p* ≤ 0.0001) of 6.5 were observed. For instance, PC_Ag0.1 sample reduced bacterial growth by 5 log (*p* ≤ 0.0001) and 6 log (*p* ≤ 0.0001) in the non-AMR resistant strains of *E. coli and P. aeruginosa*, respectively. With regards to the Gram-positive bacteria, *S. aureus* strains have proved to be more difficult to inhibit than strains of *E. faecalis*, with bacterial reductions ranging from 9.2 log for PC_Ag0.1 to 5.5 log (*p* ≤ 0.001) and 3.8 log (*p* ≤ 0.001) for PC_Ag0.3 and PC_Ag0.75, respectively.

The highest tolerance exhibited by Gram-positive bacteria (*S. aureus* and *E. faecalis*) could be attributed to their unique cell wall structure. It has been suggested that Gram-positive bacteria demonstrate a more resistive behaviour to metallic ions due to the thick peptidoglycan (PG) layer (~ 80 nm) in their cell walls, compared to the Gram-negative negatives (*E. coli* and *P. aeruginosa*), which are covered by a lipopolysaccharide layer (LPS) (~ 1–3 μm) and thinner peptidoglycan (1–3 nm)^34^. Moreover, the higher susceptibility of Gram-negative bacteria to metallic ions can be ascribed to the fact that the lipopolysaccharide layer is negatively charged^35^. The strong interaction between the lipopolysaccharide layer and the positively charged silver ions may facilitate higher ion uptake into the bacterial cell membrane, leading to intracellular damage^34^. Interestingly, AMR strains are shown as more sensitive to the Ag^+^ compared to the non-AMR. This could be attributed to the proactive use of pumps and multidrug transporters by the AMR strains. Multidrug transporters can induce resistance to antibiotics and also increase sensitivity, as shown for *E.coli*^36^. In particular, MacB and YbhFSR efflux transporters have been recently characterised as mediators to antibiotic resistance^36,37^.

A possible mechanism of action could start with the interaction between the positively charged silver ions and the negatively charged bacterial cell membrane. Upon the attachment, alterations on the cell membrane occur (i.e. membrane potential, viscoelasticity, phospholipid orientation)^38^. Such alternations modify the ionic diffusion rate and thus, stability of the proteins of the membrane which consequently would affect the membrane function. The interplay between the silver ions and bacterial membrane increase membrane permeability that causes membrane depolarization leading to cell death^39,40^. The structure of the bacterial cell wall and membrane (LPS) plays a major part in bacterial sensitivity to silver ions. However, other possible pathways have been proposed including the interaction of silver ions with sulfhydryl groups on the bacterial cell membrane that cause blockage of protein secretion and lipid biosynthesis^41,42^.

### Cell viability study

**Figure 6:**
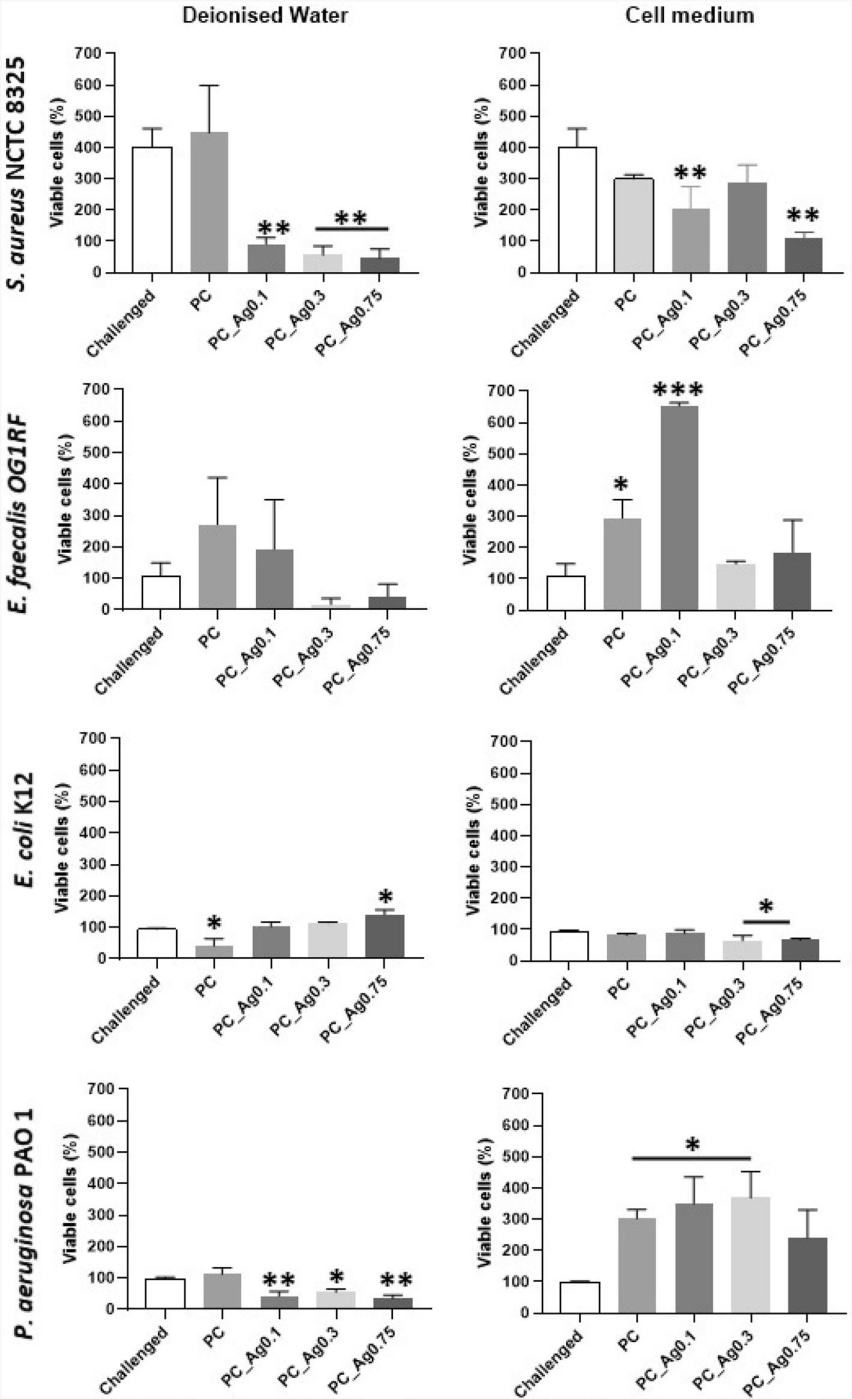
MTT analysis of HaCaT cells infected with bacteria in the presence of coacervate gels dissolution products in deionised water and cell medium. Error bars indicate the mean ± standard deviation. Statistical analysis was performed using one-way ANOVA (*p ≤ 0.05; **p ≤ 0.01; ***p ≤ 0.001). Asterisks illustrate the degree of statistical difference when compared to the challenging sample.

The cytotoxicity of the dissolution products was assessed using HaCaT cells with the addition of non-AMR strains of *S. aureus, E. faecalis E. coli and P. aeruginosa*, as illustrated in Fig. 6. Only the non-AMR strains were considered given that these strains seem to be more tolerant to the PC_Ag dissolution products than the AMR strains. Regardless of the presence of bacteria, higher cytotoxicity was observed in water than in cell medium.

Increasing concentrations of Ag^+^ trigger a negative effect on the viability of the challenged cells, especially when exposed to *S. aureus* and in water. The only exception is for the cells combined with *E. coli* since higher levels of silver resulted in a higher number of viable cells, with PC_Ag0.75 samples showing a significant increase (*p ≤ 0.05) in cell viability. In cell medium, the decrease in cell viability is only evident when using PC_Ag0.3 and PC_Ag0.75 samples. Bacteria seem to increase cell viability in combination with PC_Ag0 and PC_Ag0.1 samples, confirming no cytotoxic effects from low silver concentrations. Increasing silver concentrations normally results not only in higher bacterial inhibition^14,43^ but also in a more aggressive effect on the HaCaT cells, leading to high levels of toxicity.

These results show cytocompatibility of the dissolution products in both deionised water and cell medium. These observations are in agreement with previous studies reporting the protective role of silver on human keratinocytes^44,45^.

### Antibacterial effect on human keratinocyte (HaCaT) cells

**Figure 7:**
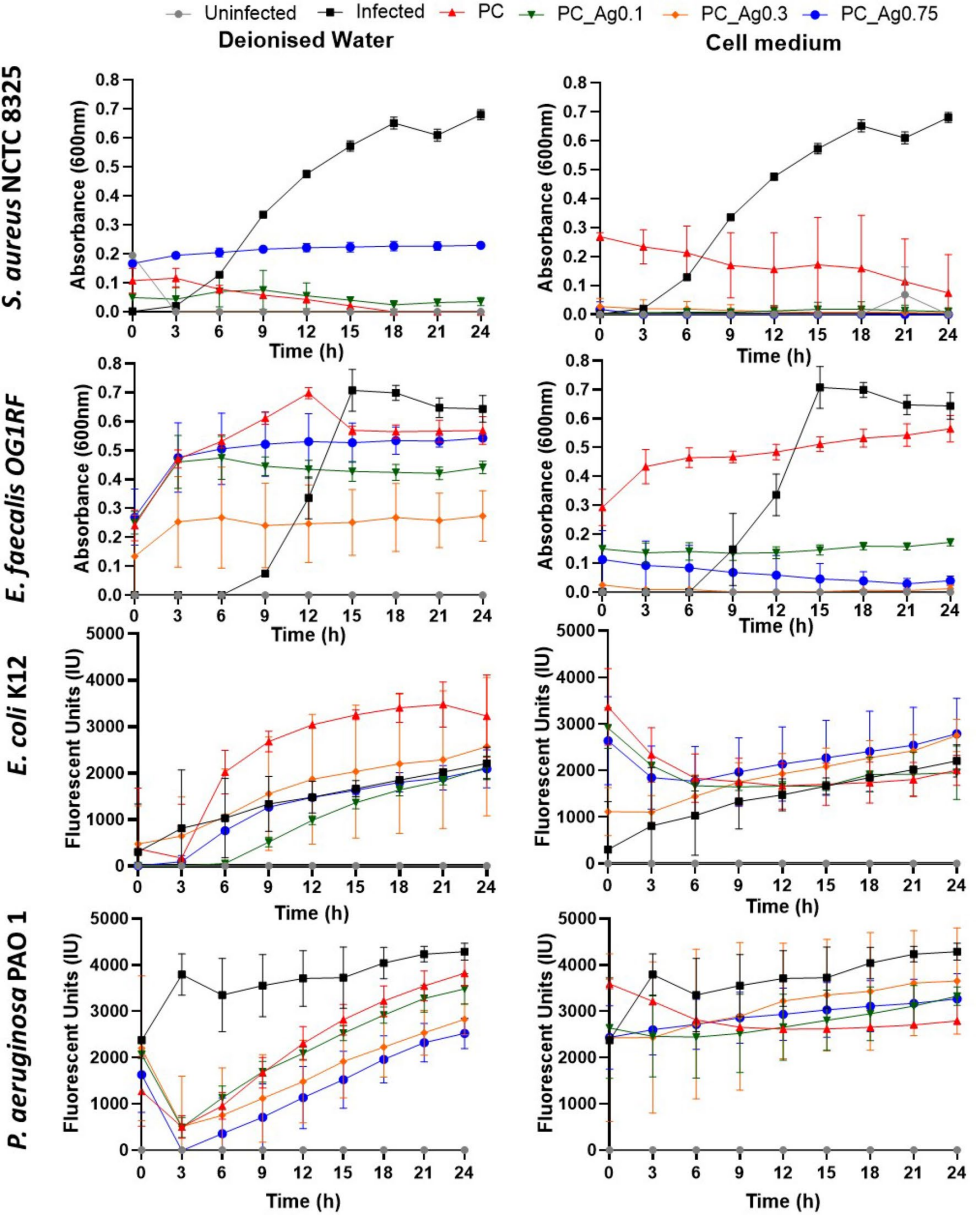
Infection of HaCaT cells with bacterial strains after 24 h incubation with coacervate dissolution products in water and cell medium. A culture of HaCaT cells was used as a control (uninfected). Error bars indicate the mean ± standard deviation (n = 3).

The antibacterial effect of PC gels was further tested against the non-AMR strains of *S. aureus*, *E. faecalis*, *E. coli* and *P. aeruginosa* when grown together with HaCaT cells (Fig. 7). Bacterial growth was measured based on absorbance (600 nm) for *S. aureus* and *E. faecalis* and fluorescent units for *E. coli* and *P. aeruginosa*. Therefore, changes in absorbance or fluorescent levels indicate fluctuation only in bacterial viability when compared to the control samples. HaCaT cells exposed only to bacteria (challenged), but not in contact with the dissolution products, were used as controls. As shown in Fig. 7, in absence of dissolution products (grey and black lines), the similarity was observed for a specific bacterium in both deionised water and cell medium, ruling out any interference from the growth environment. However, even in the absence of dissolution products, a difference was observed between different types of bacteria.

Except for *S. aureus*, PC gels dissolved in water show a significant antibacterial effect dependent on the silver content, especially against *P. aeruginosa*. It is worth noting that PC_Ag0.3 is the most effective sample against *E. faecalis*, while *E. coli* seems to be relatively sensitive to all doped PC samples. Interestingly, the presence of silver does not result in any additional effect against *S. aureus* since dissolution products with no silver can reduce bacterial growth down to the same levels observed with doped PC gels, either in water or cell medium. In the cell medium, a positive correlation between the silver content and the antibacterial activity against *E. faecalis* was also observed. However, neither undoped nor doped samples were capable of inhibiting *E. coli* and *P. aeruginosa* in cell medium, with no significant differences from the control. Results presented in Fig. 7, show that the two Gram-positive bacteria (*S. aureus* and *E. faecalis*) are much more sensitive to the PC_Ag gels that the Gram-negative (*E. coli* and *P. aeruginosa*), particularly in cell medium. These results are in agreement with a previous study that showed a 40% increase in the growth rate of Gram-negative bacteria in the presence of polyphosphates in solution if compared to the typical bacterial broth^46^.

## Conclusions

A series of silver doped PC gels were prepared via coacervation in an aqueous solution at room temperature to test (a) their antibacterial activity against a series of bacteria commonly associated with wound infections and AMR (*S. aureus, E. faecalis, E. coli *and* P. aeruginosa*) and (b) their biocompatibility against HaCaT cells. Results have shown that the PC_Ag gels have a significant antibacterial effect that is dependent on the Ag^+^ concentration, the type of bacteria tested, the environmental growth conditions and the medium used to dissolve the gels. Dissolution tests have demonstrated a controlled, time-dependent release of silver ions. In addition, pH studies suggest that in cell medium the initial pH of ~ 8 decreases only slightly over the 7 days, remaining around neutrality (pH ~ 7). Furthermore, biocompatibility studies, suggest that PC_Ag, are not toxic for the HaCaT cells, both in the presence and in the absence of bacterial strains. In particular, PC_Ag0.1 seems to be the most promising sample given that can maintain good cell viability and simultaneously display antibacterial activity. These results show that gel-like silver doped coacervates are promising multifunctional materials being able to simultaneously induce soft tissue regeneration and controlled delivery of antibacterial ions.
